# Intervention for alcohol use disorders at an HIV care clinic in Harare: a pilot and feasibility study

**DOI:** 10.1186/s13722-019-0143-7

**Published:** 2019-04-05

**Authors:** Munyaradzi Madhombiro, Bazondlile Dube, Michelle Dube, Moleen Zunza, Dixon Chibanda, Simbarashe Rusakaniko, Soraya Seedat

**Affiliations:** 1 Department of Psychiatry, College of Health Sciences, Parirenyatwa Group of Hospitals, Avondale, Zimbabwe; 2 Psychiatric Hospital, Harare Central Hospital, Southerton, Zimbabwe; 3 Global Health, Faculty of Medicine and Health Sciences, Stellenbosch University, Stellenbosch, South Africa; 4 Department of Community Medicine, College of Health Sciences, University of Zimbabwe, College of Health Sciences, Harare, Zimbabwe; 5 Department of Psychiatry, Faculty of Medicine and Health sciences, Stellenbosch University, Cape Town, South Africa

**Keywords:** Alcohol, HIV, MI/CBT intervention, Psychological, Task sharing, Zimbabwe

## Abstract

**Background:**

Alcohol use in HIV infected patients is associated with risky sexual behaviour, poor adherence to Highly Active Antiretroviral Therapy, treatment failure and increased physiologic harm. The objectives of the study were to pilot the outcome assessments to be used in the trial proper, assess the feasibility of delivery of a brief MI/CBT intervention compared to an WHO mhGAP intervention for problematic alcohol use in PLWH in Zimbabwe, and pilot the effectiveness (on alcohol use, functionality and CD4 count) of these interventions at 3 months in a randomised controlled trial design.

**Methods:**

An intervention for HIV infected patients with problematic alcohol use, developed through adaptation of existing evidence based psychological treatments, was assessed for its feasibility at a tertiary HIV care clinic in Zimbabwe. Registered general nurses, using a manualised protocol, delivered the intervention. Forty patients were recruited and randomised to receive either an MI/CBT intervention or the WHO mhGAP Intervention Guide for AUDs (n = 20 patients per group).

**Results:**

Out of 40 participants enrolled, 31 were successfully followed up for 3 months with a loss to follow-up rate of 23%. There was a statistically significant decrease in AUDIT score over time in both groups (*p* < 0.001), however no statistically significant group difference with a mean difference of 0.80, standard error of 2.07 and *p* = 0.70. For the CD4 count, the median and interquartile ranges at baseline for MI/CBT and WHO mhGAP IG groups were 218 (274) and 484 (211.50), respectively. At follow-up, median and interquartile ranges for the CD4 count for MI/CBT and WHO mhGAP IG groups were 390 (280) and 567 (378), respectively, indicative of improvement in immunological parameters in both arms.

**Conclusion:**

The findings from this pilot study suggests that a brief MI/CBT delivered by Registered General Nurses for problematic alcohol use is feasible in this population but will require the implementation of additional measures to improve retention. However, mechanisms to improve retention need special attention.

*Trial registration* Pan African Clinical Trial Registry, current PACTR201509001211149

## Background

Alcohol and illicit drug use can interfere with the success of HIV treatment programs and should be specifically targeted at patients in HIV care settings [52]. A US study found that alcohol use in HIV positive individuals was associated with higher rates of medical complications and poorer HIV treatment outcomes, including reduced viral load suppression [9]. Studies have shown that people living with HIV (PLWH) drink twice as much as the general population, with increased mortality and physiological injury occurring at relatively low levels of alcohol use [21]. Studies from Nigeria, Brazil and Ethiopia have documented prevalence rates of problematic alcohol use in PLWH in the range of 14% to 32% [15, 19, 51]. There is no data on problematic alcohol use in HIV infected persons in Zimbabwe Although the burden of disease is high with 1.6 million Zimbabweans living with HIV and 800,000 on antiretroviral treatment [38]. With this burden of HIV and with high levels of alcohol intake, a significant proportion of the population may have problematic alcohol use requiring intervention that is culturally acceptable.

A systematic review for determinants of adherence to HAART found alcohol use to be an obstacle to adherence [24]. Alcohol use is associated with Hepatitis B and C co-infection and in PLWH liver malfunction may affect the metabolism of antiretroviral drugs [46]. Aside from the problems of non-adherence in PLWH, poor treatment outcomes may result from the physiological effects of alcohol. There is evidence that interventions for alcohol use can lead to the reduction in alcohol consumption in the general population [9]. Studies have also shown that psychological interventions in PLWH can improve medication adherence even when these interventions have no effect on alcohol use [48, 54]. Motivational interviewing, cognitive behaviour therapy, problem solving techniques, contingency management and twelve-step facilitation are some of the psychological interventions that have evidence of effectiveness [11].

In developed countries where, highly trained specialists usually deliver psychological therapies, it has been shown that they can lead to a change in drinking behaviour. There have only been a few studies in low resource settings [42]. A Kenyan study by Papas et al. [39], using an adapted cognitive behavioural therapy intervention, showed improvement in alcohol use outcomes in HIV infected individuals [39]. Another study from Uganda found that counselling and motivational interviewing, had more beneficial effects in women than in men with hazardous alcohol use [54]. Another study undertaken in South Africa that utilised group therapy also documented a reduction in the amount of alcohol consumed by drinking participants [59]. A qualitative study to explore the acceptability of an alcohol use intervention by Kekwaletswe et al. found that participants were willing to engage in an alcohol-focused motivational interviewing treatment [27].

Although motivational interviewing is effective in motivating individuals to reduce alcohol consumption, its effects have been shown to fade over time and booster sessions have been recommended [41]. Further, some studies have combined motivational interviewing with cognitive behavioural therapy in participants with comorbidities, such as depression and anxiety [47]. Zimbabwe has commenced rollout of the WHO mh-GAP IG for mental health interventions [31]. This has followed a call by the WHO for wide dissemination of the WHO mh GAP IG in an endeavour to close the treatment gap for mental, neurological and substance use disorders. Moreover, Counselling for Alcohol Problems (CAP), a large study in India used WHO mh-GAP IG as a comparison in their mental health trials [36]. Use of the WHO mh-GAP IG in PLWH with problematic alcohol use, if shown to be effective, can further reduce the treatment gap for mental disorder comorbidities in this vulnerable population.

While these studies have demonstrated benefits, larger sample studies are required [39, 54]. Further, variation in selection criteria and in the type of interventions across studies makes comparability challenging [17]. For example, patients with more dependence may require a larger dose of an intervention and may also benefit from stepped up care, yet most studies focus on a single intervention [18]. To this end, there are studies being conducted to assess the effectiveness of psychological therapies in HIV in addressing mental health and drinking problems [29, 40]. Limited access to skilled staff may impede the successful implementation of interventions for the management of HIV and comorbid alcohol use [43] and there have been calls to use task sharing as a way to address the staff and skills shortage [14, 28, 42]. Given that highly skilled personnel have traditionally delivered psychological therapies it is essential to find innovative ways of bridging this gap. HIV care providers, such as nurses, could be trained in the safe and effective delivery of treatments for problematic alcohol use in PLWH and in so doing improve medication adherence and HIV outcomes [7].

Feasibility studies are necessary to assess (i) contextual factors that may need to be incorporated into treatment protocols, (ii) barriers and facilitators to the provision of interventions, (iii) service-related factors and (iv) the availability of resources to deliver the interventions as this can potentially influence outcomes [6]. This study sought to obtain baseline data on problematic alcohol use using the AUDIT in a nurse driven HIV care setting. In addition, we sought to assess the utility of the AUDIT and obtain pilot data on the effectiveness of an intervention for problematic alcohol use, as delivered by registered general nurses.

### Ethics

The Health Research Ethics Committee of the Stellenbosch University (SI14/10/222), the Medical Research Council of Zimbabwe (A/1936) and the Harare Hospital Ethics Committee approved this study.

### Aim

To pilot the outcome assessments to be used in the trial proper, assess the feasibility of delivery of a brief MI/CBT intervention compared to an WHO mhGAP intervention for problematic alcohol use in PLWH in Zimbabwe, and pilot the effectiveness (on alcohol use, functionality, quality of life and CD4 count) of these interventions at 3 months in a randomised controlled trial design.

## Methods

### Recruitment

Recruitment of participants took place from the 8th to the 28th January 2016. The final assessment of outcomes was completed on the 30th of April 2016. The study setting was a tertiary HIV clinic run by nurses and medical doctors. Research assistants recruited participants during their routine clinic attendance. Informed consent was obtained from willing participants. Baseline data were collected on all participants and the interventions were delivered in February and March 2016. A computer-generated randomization schedule was used to assign participants to the two interventions. As a retention strategy, we noted the contact details of participants and their significant others and reminded participants of their appointments on the day prior to their study visits.

### Setting

Harare Central Hospital HIV care clinic which is a tertiary level clinic. Patients were referred from acute medical wards and primary care clinics in the city of Harare and Zimbabwe as a whole.

### Inclusion and exclusion criteria

Participants were adults aged 18 years and older. In order to be included, female participants had to score at least 6 and male participants at least 7 [44], respectively, on the Alcohol Use Disorders Identification Test (AUDIT), scores that are indicative of problematic drinking (www.who.int/substance_abuse/publications/audit/en) [3]. There is evidence that low alcohol consumption in HIV positive individuals is associated with increased harm than in HIV negative adults [10]. Participants were screened for cognitive impairment with the International HIV Dementia Scale (IHDS) with those by scoring 10 or less excluded. A score of 11 and below indicated cognitive impairment. Mental illness was assessed with the Substance Abuse Mental Illness Symptom Screener (SAMISS) [55]. Positive responses to questions 8-16 are suggestive of mental illness while questions 1–7 are suggestive of alcohol and other substance use problems. The Drug Use Disorders Identification Test (DUDIT) assesses other substance use with positive cut-points of 2 or more for females and 6 or more for males [5]. Participants had to be in receipt of HIV treatment at the clinic and receiving followed-up care for at least 3 months.

### Nurse training

Ten registered general nurses were invited to participate. Training included the use of PowerPoint presentations, role-play, quizzes and assignments. Training was provided both in a classroom setting and on-site. Both interventions were manualised and 5 nurses each were trained in these interventions. The trainers included two psychiatrists, a nurse practitioner and a clinical psychologist. The training was conducted over 2 days with the first day covering Good Clinical Practice and ethical principles for both groups of nurses. However, groups were separated on Day 2 of the training with each group receiving training in either MI/CBT or the WHO mh-GAP IG.

### Quality assurance

Consent was requested and obtained from the nurses to audio-record the training and this formed the basis of supervision whereby the recordings were collectively reviewed and feedback provided in order to facilitate the acquisition of a similar level of skill among the nurses. Ten percent of the sessions that were administered and audiotaped were reviewed with the nurses and feedback was provided during supervision visits. The 7 sessions that were audio taped were reviewed at the 4 supervision sessions that were held during the study. The supervision visits were held on days and times when the clinic was closed to patients for medication collection. Where inconsistencies were noted, further training was provided, both in individual and group supervision format. Client evaluation and satisfaction cards were reviewed with each nurse, and where concerns were identified, they were discussed and resolved.

### MI/CBT intervention

The MI/CBT intervention comprised a combination of motivational enhancement and cognitive-behavioural techniques and was an adaptation of these evidence-based therapies for problematic alcohol use [45, 50]. Motivational interviewing is a therapy developed by Miller and Rollnick [33] to treat patients with problematic alcohol use. It can also be used for other problematic behaviours. Patients with unhealthy alcohol use can change their drinking patterns when sufficiently motivated through counselling that emphasizes empathic listening, avoiding arguments, rolling with resistance, and self-efficacy. Cognitive behavioural therapy (CBT) is a treatment based on the premise that certain behaviours are the result of faulty thinking. Drinking behaviour is associated with certain thinking patterns that result in cravings that can be addressed by CBT. Drinking can also be avoided if the patient has certain drink refusal skills that can be developed through CBT.

The intervention comprised 4 sessions, with each session delivered in two parts as shown in Table 1. The duration of these sessions ranged from 30 min to 1 h. Table 1 shows the content [33] and duration of sessions. The motivational enhancement therapy themes included providing personal feedback on alcohol use, ‘change talk’ themes, and readiness to change the ‘ruler’. The cognitive therapy elements encompassed setting goals, dealing with cravings and cues, faulty thinking patterns, dealing with stresses, and developing ‘drink refusal’ skills. Problem solving skills were also included in the intervention. All of the intervention sessions were audiotaped and reviewed with the intervention staff to facilitate uniformity in the delivery of the intervention.

**Table 1:** Showing the MI/CBT intervention sessions

Session 1a, 30 min	General life personal goal setting Alcohol goals General life personal goal setting How alcohol use interfere with goals
Session 1b, 30 min	Possible reasons why people drink Establish you clients’ reasons for drink Providing personal feedback moving the client towards change Explaining the implications of drinking on treatment as indicated on CD4 and viral load Meaning of improvement and the value of a functional life and quality of life Drinking problems warning signs
Session 2a, 30 min	Use MI to build rapport and develop readiness to change Assess readiness to change (using readiness ruler) Assess pros and cons of change (Decision-balance exercise) Use MI to try and shift participant Elicit a commitment to change
Session 2b, 30 min	Brief explanation and principles of CBT Explanation on what triggers, urges and cues are and how they lead to drinking Discussion on how triggers arise and how they lead to use and effects Managing thoughts about drink
Session 3a, 30 min	Triggers External Internal Drink refusals skills
	Managing situations were drinking are unavoidable
Session 3a, 30 min	Dealing with specific treatment issues like Receiving viral load and CD4 results, treatment problems, losing a spouse, employment threatened by illness, new illness related to the viral infection running out of medication
Session 4, 30 min	Planning future direction Life is back to normal: Do I drink? Dealing with anger and criticism Dealing with failure Self-referral Conclusion and planning the future

WHO mh-GAP IG: The World Health Organisation Mental Health Action Programme Intervention Guide (WHO mh-GAP IG) was the comparator [57]. The WHO mhGAP IG is designed for screening and management of common mental disorders, including problematic alcohol use, in primary care settings. It has been used as a control in other studies in low and middle-income countries [37]. The WHO mhGAP comprised an assessment of alcohol use on history, brief advice on harmful alcohol use, and referral for probable alcohol dependence. This intervention was administered in a single session of 1 h by trained RGNs.

### Assessment measures

At baseline and follow-up, the AUDIT, CD4 count, WHODAS 2.0 and the WHOQOL were administered. Follow-up assessments were conducted for both group at 3 months.

#### WHO AUDIT

The WHO AUDIT was used to screen participants and was also the primary outcome measure. The AUDIT is a 10-item tool with a score range of 0 to 40 [8, 49]. In this study, scores of 6 and 7 for females and males, respectively, were applied as cut-offs to identify patients with problematic drinking based on psychometric performance of the AUDIT in previous studies [44]. Some studies have shown that HIV infected individuals require much less alcohol to get intoxicated and suffer physiologic harm [26, 30]. Although the AUDIT has not been validated in Zimbabwe or among people living with HIV, it has been used in diverse population groups in Southern Africa and in Zimbabwe [12, 13]. The AUDIT has good psychometric properties with a Cronbach alpha of 0.93 [95% CI (0.921–0.941)], specificity of 89.6% (95% CI 76.11–96.02 and sensitivity of 95.07% (95% CI 92.18–96.97) against gold standard DSM-IV criteria, at a cut point of 6 [44]. The Cronbach alpha for the AUDIT in this study was 0.72.

#### Viral load and CD4

Trained general nurses collected blood for baseline viral loads and CD4 count. These assays were done at the hospital laboratory. As a result of budgetary constraints, viral loads were not done at the 3-month assessment.

#### World Health Organisation Disability Assessment Schedule 2.0 (WHODAS 2.0)

The WHODAS 2.0 is a tool developed by the WHO to assess the degree of disability and has been validated for a variety of health conditions ([22]; www.who.int/icidh/whodas/; www.who.int/mental_health/media/en/613.pdf) [56]. Although the WHODAS 2.0 has not been validated in Zimbabwe, it has been used in rural Ethiopia and was found to have good psychometric properties with an internal consistency ranging from very good to excellent (Cronbach’s alpha 0.82 to 0.98) [23]. The WHODAS 2.0 has been validated for chronic diseases, such as HIV, and had good psychometric features [22]. The Cronbach alpha for the WHODAS 2.0 in this study was 0.91.

#### World Health Organisation Quality of Life HIV (WHOQOL HIV)

Quality of life was assessed using the WHO Quality of Life HIV (WHOQOL HIV) that has been validated for HIV elsewhere but not in Zimbabwe ([58, 25]; www.who.int/mental_health/media/en/613.pdf). The WHOQOL HIV has been validated in South Africa and Zambia and has good internal consistency with Cronbach alphas ranging from 0.889 to 0.933 in the Zambian study [35, 53]. The Cronbach alpha for the WHOQOL HIV in this study was for 0.9 D for domain I, 0.59 for domain II, 0.92 for domain III, and 0.49 for domain IV.

### Data analysis

Data were analysed using SPSS. A mixed model ANOVA was used in the analysis. Primary and secondary outcomes were measured at two time points at baseline and 3 months. The standardized mean difference of the AUDIT score was the measure of treatment effect. We summarized continuous variables using means (standard deviation) or medians (interquartile range) depending on the distribution, and categorical variables using counts (percentages). We used t-tests or Mann–Whitney U tests to compare means or medians between the two intervention groups. We used Chi square tests or Fisher’s exact tests to test the association between study groups and other categorical variables. Alpha was set at = 0.05 and all tests were two sided.

## Results

### Recruitment, randomisation and retention

**Fig. 1:**
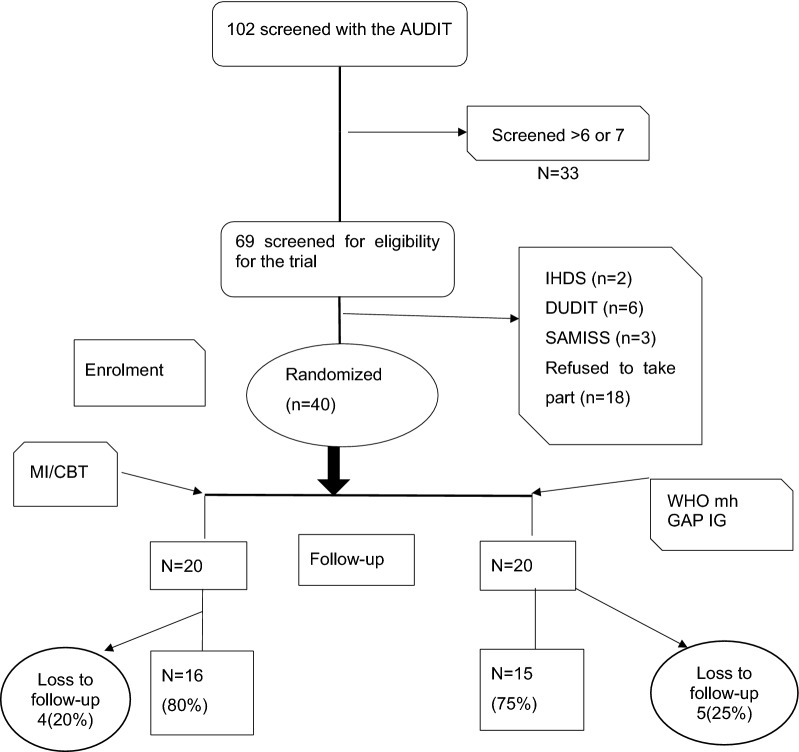
Study flow from screening to analysis

One hundred and two (102) patients were invited to participate in the study. At screening, 33 scored less than 6 on the AUDIT and 69 had scores at or above the cut-off. Eleven participants failed to meet the eligibility criteria and 18 declined to participate for reasons including moving away from Harare, work commitments and other reasons. Forty participants were randomised to each group with no baseline differences between the groups, as shown in Table 3. At follow up, 4 participants were lost in the MI/CBT group and 5 in the mhGAP IG group. Three participants in the MI/CBT group and 4 in the mhGAP IG group relocated from Harare, while 1 participant in the MI/CBT group and 1 in the WHO mhGAP IG group were unable to secure time off from work (see Fig. 1).

### Baseline characteristics

There were 17 female and 23 male participants in the study. The combined mean age was 39.5 years (SD = 9.59), with a mean age of 41.7 years (SD = 9.61) in the MI/CBT group and a mean age of 37.3 years (SD = 9.29) in the mhGAP IG group. Table 2 shows the socio-demographic characteristics of the sample. At study entry, 6 (15%) participants were on second line HAART and 34 (85%) had achieved viral suppression. Participants had been on HAART for 3.5 (SD1.2) years. The MI/CBT group had a significantly lower median CD4 count 201.5 (range of 85 to 367) than the mhGAP group 390 (range of 200 to 400) (*p* = 0.03). Table 2 shows the sociodemographic features of the study population. The participants were predominantly male and more than 50% were married.

**Table 2:** Sociodemographic characteristics of the study population

	MI/CBT	mh GAP IG
Age	39.5 (4.3)	40.1 (3.8)
*Gender*		
Female	9 (22.5%)	8 (20%)
Male	11 (27.5%)	12 (30%)
*Marital status*		
Single	4 (10%)	3 (7.5%)
Married	12 (30%)	9 (22.5%)
Divorced	2 (5%)	4 (10%)
Widowed	3 (7.5%)	3 (7.5%)
*Education*		
Years in school	9.5 (3.2)	10.1 (3.8)

### Interventions

A total of 74 interventions sessions were administered with a median of 3.7 (1 to 4 sessions) sessions per participant and 14.8 sessions per nurse. The duration of sessions ranged from 30 to 55 min. In the mhGAP group, each nurse conducted a single session of the mhGAP at the first contact. A total of 20 sessions were administered to this group.

### Outcomes

#### Alcohol use outcomes

**Fig. 2:**
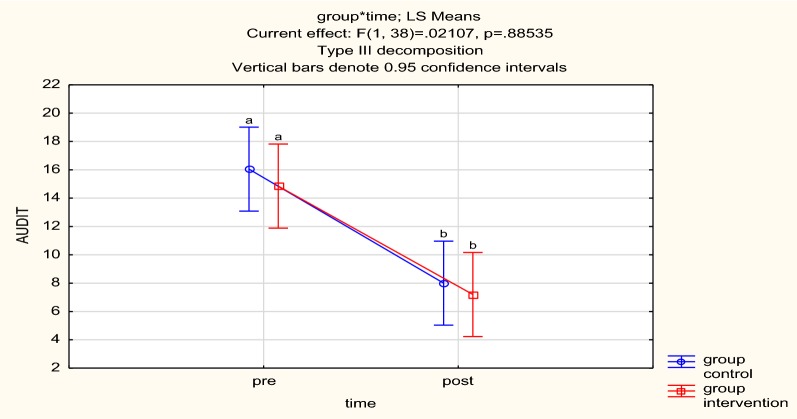
AUDIT for both arms. Both arms recorded significant falls in their AUDIT score

There was no statistically significant difference in the AUDIT score at baseline (*p* = 0.57) between the groups as shown in Table 3. There was a statistically significant change in alcohol use in both groups over time (*p* < 0.001) as shown on Table 3. There was no difference in the magnitude of change between the groups as shown the 95% CI in Fig. 2.

**Table 3:** Change in AUDIT scores between MI/CBT and mhGAP at baseline and 3 months

Arms	Mean baseline (SD)	Mean at 3 months (SD)	*p*-value
mhGAP time	16.05 (7.21)	8.00 (5.79)	< 0.001
MI/CBT over time	14.85 (7.78)	7.20 (5.07)	< 0.001

There were however no statistically significant differences in AUDIT score between the arms at baseline (*p* = 0.57) and 3 months (*p* = 0.70)

#### CD4

Due to the skewness in the data, we report medians and interquartile ranges. At follow-up, the median and interquartile ranges for the MI/CBT and mhGAP IG groups were 390 (280) and 567 (378), respectively, indicative of improvement in immunological parameters although groups differences were not statistically significant as shown in Table 4.

**Table 4:** Change in CD4 over the 3 month follow up period

	MI/CBT median	MI/CBT IQR	MH GAP IG median	MH GAP IQR	*p*-value
CD4 count baseline	218	274	484	211.5	0.85
CD4 counts follow-up at 3 months	390	280	567	378	0.56

#### Functioning and quality of life

**Fig. 3:**
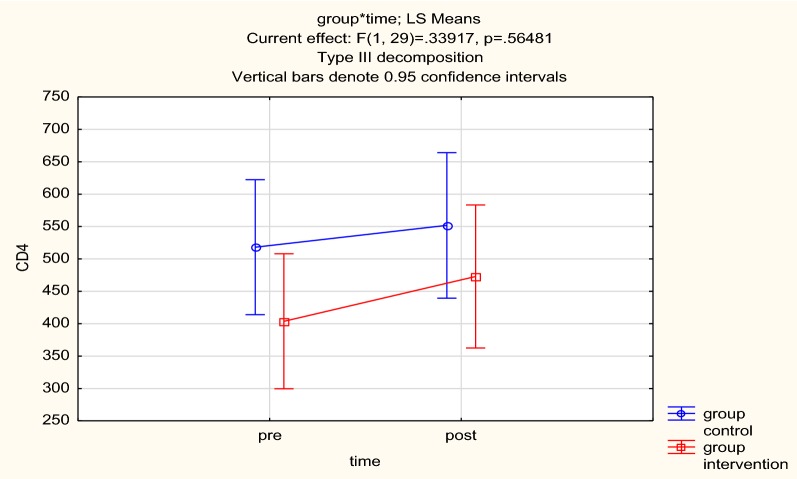
CD4 count for both arms. Group control represents the mh GAP IG arm and group intervention represents the MI/CBT arm

Functioning and quality of life, as measured on the WHODAS 2.0 and the WHOQOL HIV, showed no change over time. Tables 5 and 6 show the treatments effects on the WHODAS 2.0 and WHOQOL HIV respectively (Fig. 3).

**Table 5:** WHODAS scores for baseline and 3 months

Time-point	MI/CBT mean (SD)	MhGAP mean (SD)	*p*-value
Baseline	13.7 (6.88)	15.5 (6.48)	0.91
3 months	13.4 (3.80)	13.3 (4.35)	0.77

**Table 6:** WHOQOL (quality of life) for baseline and 3 months

Time-point	MI/CBT mean (SD)	MhGAP mean (SD)	*p*-value
Baseline	71.65 (8.07)	69.63 (7.71)	0.78
3 months	73.33 (7.11)	72.94 (8.70)	0.81

## Discussion

The aim of this study was to pilot the outcome assessments to be used in an adequately powered trial, assess the feasibility of delivery of a brief MI/CBT intervention compared to an WHO mhGAP intervention for problematic alcohol use in PLWH in Zimbabwe, and assess their preliminary effectiveness using a task-sharing model in a low resource setting with a huge HIV burden. This sample of 40 HIV infected patients was drawn from an HIV care setting at a tertiary institution where participants received either blended motivational interviewing/CBT or the WHO MH GAP IG. The retention rate was 77% at 3 months with no statistically significant group difference. In both groups, drinking behaviour as assessed by AUDIT score and CD4 count improved after treatment.

Improvement in alcohol use in this population supports previous findings that participation in screening and exposure to alcohol counselling, and receipt of pamphlets or presentations can lead to reduction in alcohol use [11]. Studies have shown that in participants with early and low levels of alcohol use, low intensity interventions can lead to change in the level of alcohol use [2]. However, studies have also shown that patients who are dependent on alcohol do not change their drinking behaviour even with longer exposure to interventions [34]. Although we hypothesized that MI/CBT would result in a greater reduction in drinking than the mhGAP IG, this was not the case. Both interventions could be considered as ‘active’ treatments. There has been a call to adapt and pilot treatments such as the WHO MH GAP IG, that are scalable in low- and middle- income countries [4].

Although there was change in the CD4 count in both groups, this was not statistically significant. Some studies have found no relationship between alcohol use and CD4 count [1]. Functional capacity and quality of life did not show any change over time in the trial. The small sample size may have contributed to the lack of detection of a significant effect.

The loss to follow up was 23% despite retention efforts such as confirming appointments. Follow up assessments did not always coincide with scheduled clinic visits. Participants at the clinic at which recruitment took place (a tertiary institution) are poorly adherent, have late stage disease and high rates of treatment failure. These are some of the characteristics of loss to follow up that were documented in a study in Ethiopia [32].

The study provides preliminary evidence that registered general nurses can be trained to offer a psychological intervention for dually diagnosed adults with HIV and unhealthy alcohol use. In Zimbabwe, nurses are at the forefront of provision of both curative and preventive interventions in Zimbabwe. While task sharing an intervention for unhealthy alcohol use to nurses may be novel, nurses have been at the forefront of provision of HIV treatment with results that are comparable to those achieved by doctors [20].

This pilot study has a number of limitations. It is characterized by a small sample and, as such, not adequately powered to demonstrate true treatment differences and hence prone to Type I error. The study recruited participants who had a known adherence, given that they were the inclusion criteria required the adherence clinic visits in the past 3 months. Further, the alcohol screening used the AUDIT, which is a self-report tool. As a result, has social desirability bias [16]. The study was undertaken at a tertiary institution which serves as a referral centre for patients and not in primary or district care settings, thus morbidity present in the sample may not be reflective of HIV patients receiving care in the latter settings.

## Conclusion

This study demonstrates the feasibility of administering the assessment tools employed here and delivering a brief MI/CBT intervention in HIV care clinics. The interventions led to change in alcohol use. Further large samples studies that are adequately powered are needed. Strategies to retain participants need to be carefully considered when integrating interventions for alcohol use treatment into usual care.

## Abbreviations

AUDIT: Alcohol Use Disorders Identification Test
AUDs: Alcohol Use Disorders
CAP: Counselling for Alcohol Problems
CBT: Cognitive Behavioural Therapy
DSM IV: Diagnostic Statistical Manual Version 4
DUDIT: Drug Use Disorders Identification Test
HAART: Highly Active Antiretroviral Therapy
HIV: Human Immunodeficiency Virus
IHDS: International HIV Dementia Scale
MI/CBT: Motivational Interviewing/Cognitive Behavioural Therapy
MhGAP IG: Mental Health Gap Action Program Intervention Guide
PLWH: People Living With HIV
RGNs: Registered General Nurses
SAMISS: Substance Abuse Mental Illness Symptom Screener
SPSS: Statistical Package for Social Sciences
WHO: World Health Organisation
WHODAS: World Health Organisation Disability Assessment Schedule
WHOQOL: World Health Organisation Quality of Life
